# Safety and Biodistribution of an Autologous Bone Marrow-Derived Mononuclear Cell Infusion into Renal Arteries in Patients with Focal Segmental Glomerulosclerosis: A Phase 1 Study

**DOI:** 10.1155/2024/2385568

**Published:** 2024-07-09

**Authors:** Bruno Freire Botelho, André Luis Barreira, Marcio Gomes Filippo, Karina Dutra Asensi, Lanuza A P Faccioli, Anna Beatriz dos Santos Salgado, Elizabeth Figueiredo de Salles, Carlos Eduardo Cruz Marques, Pedro Leme Silva, Regina Coeli dos Santos Goldenberg, Angelo Maiolino, Bianca Gutfilen, Sergio Augusto Lopes de Souza, Maurilo Leite Junior, Marcelo Marcos Morales

**Affiliations:** ^1^ Department of Nephrology Clementino Fraga Filho University Hospital Federal University of Rio de Janeiro, Rio de Janeiro, Brazil; ^2^ Department of Vascular Surgery Clementino Fraga Filho University Hospital Federal University of Rio de Janeiro, Rio de Janeiro, Brazil; ^3^ Cellular and Molecular Cardiology Laboratory Carlos Chagas Filho Biophysics Institute Federal University of Rio de Janeiro, Rio de Janeiro, Brazil; ^4^ Department of Radiology Clementino Fraga Filho University Hospital Federal University of Rio de Janeiro, Rio de Janeiro, Brazil; ^5^ Laboratory of Pulmonary Investigation Carlos Chagas Filho Biophysics Institute Federal University of Rio de Janeiro, Rio de Janeiro, Brazil; ^6^ Precision Medicine Research Center Carlos Chagas Filho Institute of Biophysics e Brazilian Institute of Science and Technology—INCT REGENERA, Rio de Janeiro, Brazil; ^7^ Department of Hematology Clementino Fraga Filho University Hospital Federal University of Rio de Janeiro, Rio de Janeiro, Brazil; ^8^ Laboratory of Cellular and Molecular Physiology Carlos Chagas Filho Biophysics Institute Health Sciences Center Federal University of Rio de Janeiro, Rio de Janeiro, Brazil

## Abstract

Patients with focal segmental glomerulosclerosis (FSGS) who are refractory to drug treatment may present progressive loss of kidney function, leading to chronic kidney disease stage 5 under dialysis treatment. The safety of systemic administration of bone marrow-derived mononuclear cells (BMDMCs) has been shown in different preclinical models of kidney diseases. However, to date, no study has evaluated the safety and biodistribution of BMDMCs after infusion in renal arteries in patients with FSGS. We used a prospective, non-randomized, single-center longitudinal design to investigate this approach. Five patients with refractory FSGS and an estimated glomerular filtration rate (eGFR) between 20 and 40 ml/min/1.73 m^2^ underwent bone marrow aspiration and received an arterial infusion of autologous BMDMCs (5 × 10^7^) for each kidney. In addition, BMDMCs labeled with technetium-99m (^99m^Tc-BMDMCs) were used to assess the biodistribution by scintigraphy. All patients completed the 270-day follow-up protocol with no serious adverse events. A transient increase in creatinine was observed after the cell therapy, with improvement on day 30. ^99m^Tc-BMDMCs were detected in both kidneys and counts were higher after 2 hr compared with 24 hr. The arterial infusion of BMDMCs in both kidneys of patients with FSGS was considered safe with stable eGFR at the end of follow-up. This trial is registered with NCT02693366.

## 1. Introduction

Focal segmental glomerulosclerosis (FSGS) is a glomerular disease caused by podocyte injury, leading to loss of integrity of the glomerular filtration barrier with proteinuria. Nephrotic syndrome is the most common clinical presentation of the primary form, and the prevalence seems to be increasing worldwide [1, 2, 3]. Immunosuppressive agents and inhibitors of the renin-angiotensin system are the main treatments, but patients who are refractory to that may progress to chronic kidney disease stage 5 treated by dialysis (CKD G5D) or kidney transplantation [4]. However, the disease recurs in the renal allograft in more than 30% of patients with primary FSGS after kidney transplantation [5].

Preclinical studies of stem cell therapy in different models of kidney diseases showed a reduction in the development and progression of CKD, including experimental FSGS, by reducing glomerulosclerosis by limiting podocyte loss and apoptosis [6]. In addition, a recent meta-analysis from clinical trials involving intravascular stem cell infusion showed a favorable safety profile [7]. Bone marrow cells are easily obtained for autologous transplantation and remain both phenotypically and functionally active in patients with CKD G5D compared with healthy controls, preserving the potential for clinical application for kidney diseases [8].

Despite recent advances in cell therapy, relevant issues must be addressed to allow proper translation from preclinical studies into clinical practice. Information on the different viable routes for the infusion, the cell source, dosage, and the biodistribution is essential to achieve the most effective cell therapy [9]. There are few studies *in vivo* with clinical cell tracking and none associated with kidney disease [10].

We tested the hypothesis that renal artery infusion of autologous BMDMCs in patients with refractory FSGS would be feasible and safe. We also analyzed the biodistribution of autologous BMDMCs labeled with technetium-99m (^99m^Tc-BMDMCs) 2 hr and 24 hr after cell therapy.

## 2. Materials and Methods

A prospective, non-randomized, single-center longitudinal study was conducted to evaluate the feasibility and safety of renal artery infusion of BMDMCs in patients with refractory FSGS and CKD. Patients were enrolled at the Clementino Fraga Filho University Hospital (HUCFF) of the Federal University of Rio de Janeiro (UFRJ), Brazil, or referred from other institutions between 15 June 2015 and 17 June 2017. The diagnosis of FSGS was established by clinical history and renal biopsy. All patients were treated with nephroprotective medication by angiotensin-converting enzyme (ACEi) inhibitor or angiotensin receptor blockers (ARBs) and without immunosuppressive therapy. Informed consent was obtained from each patient, and the study protocol was approved by the Brazilian National Ethical and Research Committee/CONEP (study ID approval: CONEP 505.871) and registered at Clinical-Trials.gov (identifier: NCT02693366). In addition to autologous infusion of BMDMCs, the patients received standard care according to the current guidelines of the Brazilian Society of Nephrology and Kidney Disease and Improving Global Outcomes.

### 2.1. Population

Five patients were studied 90 days before and 270 days after cell transplantation, completing 1 year of follow-up. The patients met the following inclusion criteria: (1) age between 18 and 60 years; (2) primary FSGS previously treated with corticosteroids and/or immunosuppressants without a satisfactory response; (3) estimated glomerular filtration rate (eGFR) using the CKD-EPI (Chronic Kidney Disease Epidemiology Collaboration) formula between 20 and 40 ml/min/1.73 m^2^; (4) use of an ACE inhibitor or ARB; (5) signed a consent form. The exclusion criteria were as follows: (1) active urinary infection; (2) urinary tract infection with tuberculosis or fungus; (3) blood pressure >160 mmHg (systolic) and 100 mmHg (diastolic) during the last three outpatient visits; (4) use of iodinated contrast in the past 3 months; (5) use of nephrotoxic drugs; (6) glucocorticoid therapy or corticosteroid therapy >0.3 mg/kg/day; (7) impossible to obtain vascular access for a percutaneous procedure; (8) sepsis criteria according to the Society of Critical Care Medicine and American College of Chest Physicians (1992); (9) malignant neoplasms; (10) autoimmune diseases; (11) neurodegenerative diseases; (12) acute or decompensated heart failure; (13) primary hematologic diseases; (14) osteopathies that reflect an increased risk of spinal puncture (15); coagulopathies (16); liver failure; (17) stroke or acute myocardial infarction in the last 6 months; (18) pregnancy or breastfeeding; (19) chronic infectious diseases; and (20) participation in another clinical trial in the last year.

### 2.2. Bone Marrow Aspiration, Cell Separation, and Labeling with Technetium-99m

On the day of the injection procedure, 180 ml of bone marrow was aspirated from each patient under spinal anesthesia from the posterior iliac crest. BMDMCs were isolated by density gradient centrifugation at 400 × *g* for 30 min (Ficoll-Paque Plus 1.077, 1 : 2, Amersham Biosciences, São Paulo, Brazil). Mononuclear cells were washed in saline containing 5% human serum albumin and filtered through a 100-*μ*m nylon mesh to remove cell aggregates. After washing, counting, and viability testing, the cells were resuspended in 10 ml of saline solution with 5% autologous serum. For each patient, 2 × 10^7^ cells were labeled with ^99m^Tc based on previous studies [11]. The cell preparation and labeling procedures were carried out in a laminar flow. Briefly, 500 *μ*l of sterile SnCl_2_ solution was added to the cell suspension in 0.9% NaCl, and the mixture was incubated at room temperature for 10 min. Then, 45 mCi of ^99m^Tc was added and the incubation continued for another 10 min. After centrifugation (500 × *g* for 5 min), the supernatant was removed and the cells were washed again in saline solution. The pellet was resuspended in saline solution. The viability of the labeled cells was assessed by the trypan blue exclusion test and was estimated to be >93% in all cases. Labeling efficiency (%) was calculated by the activity in the pellet divided by the sum of the radioactivity in the pellet plus supernatant and was estimated to be >90% in all cases. Bacteriologic analyses and cultures were also carried out to exclude contamination of the material. A sample of the isolated BMDMCs was characterized by flow cytometry analysis of specific surface antigens as previously described [12].

### 2.3. Flow Cytometry Analysis

The samples were processed by flow cytometry to identify populations of BMDMCs. Cells were incubated with specific antibodies for 20 min in the dark at room temperature. Specific surface antigens conjugated with fluorochromes were assessed using the following antibody combinations: CD45 FITC (clone HI30; BD Biosciences), CD13 PE (clone WM15; BD Pharmingen) and CD11b APC (clone MEM-174; Exbio) to neutrophils; CD34 FITC (clone 8G12; BD Biosciences), CD117 PE (clone YB5.B8; BD Pharmingen), HLA-DR PE-Cy5 (clone TU36; BD Pharmingen) and CD45 APC (clone MEM-28; Exbio) to hematopoietic progenitor cells; CD64 FITC (clone10.1; BD Pharmingen), CD34 PE (clone 8G12; BD Biosciences), CD14 PE (clone M*φ*P9; BD Pharmingen) and CD45 APC (clone MEM-28; Exbio) to promonocytes and monocytes; CD20 FITC (clone LT20; Exbio), CD10 PE (clone MEM-78; Exbio), CD19 APC (clone HIB19; BD Pharmingen) and CD45 APC-Cy7 (clone 2D1; BD Pharmingen) to B-lymphocytes; lineage cocktail 2 FITC (LIN2 was composed of CD3, CD14, CD19, CD20 and CD56) (clones SK7, M*φ*P9, SJ25C1, L27, NCAM16.2, respectively; BD Biosciences); CD105 PE (clone 266; BD Pharmingen), CD90 PE-Cy5 (clone 5E10; BD Pharmingen), CD73 APC (clone AD2; BD Pharmingen) and CD45 APC-Cy7 (clone 2D1; BD Pharmingen) to mesenchymal cells; Lymphogram (composed of CD8/CD19 FITC, CD3/CD56 PE, CD4 PE-Cy5) (clones UCH-T4, HD37, 33-2-A3, C5.9, 13B8.2; Cytognos) and CD45 APC (clone MEM-28; Exbio) to a subpopulation of lymphocytes (cytotoxic T cell, Pan B cell, Pan T cell, NK cell, helper T cell, respectively), and CD36 PE (clone CB38; BD Pharmingen), CD71 APC (clone M-A712;,BD Pharmingen) and CD45 FITC (clone HI30; BD) to erythrocytes population. The lysis of erythrocytes was performed using FACS lysing solution (BD Biosciences). Data were acquired on a BD FACS ARIA II (BD Biosciences) flow cytometer and analyzed using Infinicity software (Cytognos, Spain).

### 2.4. Renal Arterial Infusion

After 4 hr of bone marrow aspiration, the patient was referred to the Hemodynamics Service for the endovascular procedure. Cells labeled as described above were added back into the total mononuclear cell suspension (final volume of 10 ml) and then divided into two samples of 5 ml each. The right femoral artery was punctured under local anesthesia by inserting a 5-Fr 11-cm sheath. Patients underwent anticoagulation with intravenous heparin to obtain an activated clotting time of two to three times baseline. A 5-Fr guiding catheter (Simons 1; Envoy—Cordis, Miami, FL) and a 0.889-*μ*m (0.035-inch) hydrophilic guide wire (Roadrunner; Cook Medical) were passed through the sheath to cannulate the renal arteries. Digital abdominal arteriography was performed (Angiostar; Siemens Medical Systems, Erlangen, Germany) to allow visualization and catheterization of the renal vasculature before injection and to monitor flow normality and vessel patency. A 5-ml aliquot of the autologous BMDMC solution with about 5 × 10^7^ cells was infused slowly into each kidney. To prevent nephrotoxicity from the iodinated contrast, the protocol was performed with bicarbonate solution; 3 ml/kg 1 hr before and 1 ml/kg for 6 hr after the procedure associated with 600 mg n-acetylcysteine 12 hr/12 hr orally 24 hr before and after implant of the aspirate. The minimum possible amount of non-ionic iodinated contrast of low osmolarity (iopamidol) was used to visualize the renal arteries. After the endovascular procedure, patients were monitored for 1 hr and sent to the Nuclear Medicine Department for examination.

### 2.5. Imaging Analysis

Whole-body and planar images were acquired using a Millennium GE gamma camera (General Electric Medical Systems, Milwaukee, WI). Acquisition protocols were performed at 2 hr and 24 hr after cell therapy. Whole-body images were acquired for 20 min in anterior and posterior views, using a dual-head whole-body scanner with a high-resolution and low-energy collimator. Planar images were acquired for 10 min, matrix 256 × 256, in anterior, right and left lateral, and posterior views. Single-photon-emission computed tomography (SPECT) was performed for 24 min with two 180° opposed rotating detectors and low-energy high-resolution collimators. The software and hardware for reconstruction of the SPECT image data was a Xeleris-GE processing workstation. For each patient, regions of interest were drawn for the kidney, liver, spleen, lungs, bladder, and brain, and for the whole body, and the radioactive counts were automatically quantified for these regions in whole-body planar images at 2 and 24 hr after cell injection. Uptake was defined as the percentage of organ-originated counts compared with the total number of counts in the whole body. Computed tomography (CT) scans were acquired using an Optima 560 PET/CT scanner (GE Healthcare, Boston, MA). SPECT and CT images were exported as DICOM to a Macintosh computer (MacOS X, version 10.4). Images were fused with dedicated image processing software (OsiriX imaging software, version 3.6; Geneva, Switzerland).

### 2.6. Clinical and Laboratory Variables

Physical examination, medical history, and vital signs were assessed before and after the cell therapy at −90, −45, −7, 0, +1, +2, +7, +15, + 30, +90, +180, +270 days. Laboratory tests, renal scintigraphy, and the SF-36 Quality of Life questionnaire [13] were executed as described. Imaging and cardiology examinations (electrocardiogram, transthoracic echocardiogram, chest radiography, ultrasonography of the total abdomen and urinary tract, and Doppler ultrasonography of renal arteries) were assessed at −90 and +270 days (Table S1). The patients were hospitalized 1 day before cell transplantation and remained in hospital for 48 hr after the procedure. The patients received granulocyte colony-stimulating factor (G-CSF; 5 *μ*g/kg/body weight) daily for 5 days (maximum dose 300 *μ*g) about 30 days after cell therapy to increase the bone marrow mononuclear cells released into the circulation.

### 2.7. Statistical Analysis

The Kolmogorov–Smirnov test with Lilliefors' correction was used to test for the normality of the data distribution; the Levene median test was used to evaluate the homogeneity of variances. Laboratory data obtained from blood and urine samples at −30, −7, +1,+7, + 15, + 30, +90, +180 e + 270 days were analyzed by repeated measures Friedman test followed by Dunn's multiple comparisons test (*p* < 0.05). Laboratory data obtained from blood samples at −90 days and 270 days after bone marrow therapy were analyzed by paired Wilcoxon test (*p* < 0.05). The single imputation method of regression substitution for missing data for two patients at one time point in the biochemical analysis and urine protein content analysis at 360 days was used. Parametric data are expressed as means ± standard deviation and nonparametric data are expressed as the median (interquartile range). The GraphPad Prism statistical software package (version 8.3.1; GraphPad, La Jolla, CA) was used. A *p* value < 0.05 was considered significant. The primary endpoint focused on safety and included death and deterioration in renal function.

## 3. Results

### 3.1. Clinical Evaluation

Five patients were enrolled in the study. They completed the follow-up assessment for 12 months, with 100% survival and clinical stability, and no losses or exclusions. The patients' characteristics at entry are shown in Table 1. The mean age at the beginning of follow-up was 41 years (range, 32−56 years), the mean urine protein-creatinine ratio (UPCR) was 1591 mg/g (range, 214−3340 mg/g), and the mean eGFR was 30.8 ml/min/1.73 m^2^ (range, 24–37 ml/min/1.73 m^2^). Nephrotic syndrome was the most common clinical presentation and all these patients had been treated previously with immunosuppressants, suggesting primary FSGS. Only patient 4 had a reported histologic variant of FSGS (collapsing), considered to have the worst prognosis. There was no clinical or laboratory evidence of secondary causes of FSGS, such as drugs, toxins, and viral infections, nor a family history of proteinuria or onset of the disease in childhood or early adult life, suggesting genetic FSGS. No patient received immunosuppressive therapy throughout the entire follow-up period, maintaining only supportive measures, such as inhibition of the renin–angiotensin system.

Patients were hospitalized 1 day before the procedure and remained for 48 hr after the endovascular procedure without any change in viable signals. The clinical events and treatments that occurred during follow-up are shown in Table S2. Patient 5 presented symptoms of pyelonephritis and was treated with ciprofloxacin for 7 days approximately 2 months after cell therapy (day +60). Patient 2 was diagnosed with pharyngitis and was treated with azithromycin after cell therapy (day +180). Patient 4 presented phlebitis during hospitalization and had acute renal colic at day +270 treated with symptomatic medication. Patient 1 was treated with topical medication due to cutaneous herpes at day +180. Patient 3 had symptoms suggestive of arbovirus at day +180 evolving with spontaneous improvement. The filgrastim protocol (G-CSF) was well tolerated; the only report of bone pain was in patient 2 who was medicated with analgesics. Imaging and cardiology examinations were performed at the beginning and end of follow-up (−90 and +270 days) with no evidence of structural and/or neoplastic changes. Figure S1 illustrates the progression of the quality of life domains during the follow-up period, as measured by the SF-36 questionnaire. These parameters remained stable throughout the observation period.

### 3.2. Cell Infusion

The bone marrow aspiration and endovascular procedures were well tolerated without significant complications. The infusion of BMDMCs did not present adverse reactions, and there was no evidence of infectious, neoplastic, or thromboembolic events. One patient presented hematoma at the puncture site due to bone marrow aspiration; four patients presented hematoma related to the endovascular procedure. In all cases, treatment with cold compresses and analgesics was sufficient. Catheterization of the left renal artery was difficult in patient 3 due to the presence of an anatomic variation (accessory artery) with vascularization of 50% of the organ, which was not detected on EcoColor Doppler performed at the beginning of the study. The characteristics of each patient's bone marrow aspirate and the volume of contrast used are listed in Table S3.

### 3.3. Imaging

**Figure 1:**
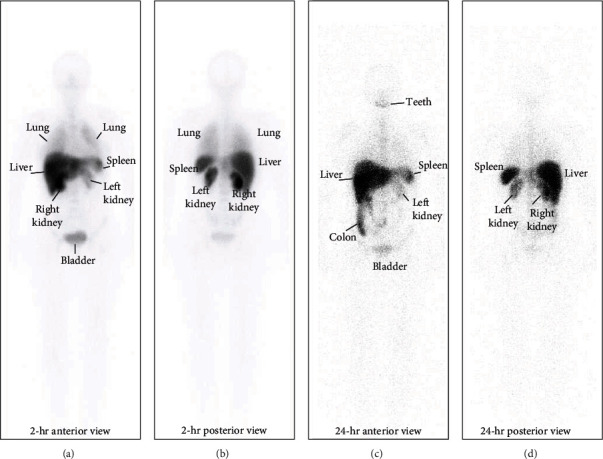
Representative image of whole-body scintigraphy showing the biodistribution of ^99m^Tc-labeled BMDMCs in patient 2 at 2 and 24 hr after cell infusion. Anterior (a) and posterior (b) view of whole-body scintigraphy 2 hr after cell administration. Anterior (c) and posterior (d) view of whole-body scintigraphy 24 hr after cell administration.

**Figure 2:**
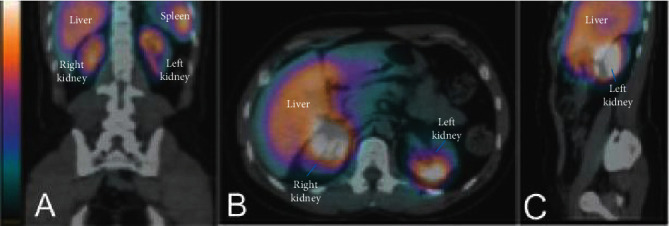
Representative images of SPECT/CT fusion in patient 2 at 2 hr after the infusion of ^99m^Tc-labeled BMDMCs showing uptake in the renal topography through different views: (A) coronal, (B) axial, and (C) sagittal. Increasing uptake is indicated by a color shift from blue to red to yellow to white.

Imaging findings are shown in Figures 1 and 2.  Figure 1 is a representative whole-body image of the heterogeneous distribution of the labeled cells 2 hr after intra-renal arterial infusion, with clear uptake in the kidney, bladder, liver, and spleen.

Figure 2 is a representative image of SPECT/CT fusion 2 hr after cell therapy showing uptake in the renal topography. The quantification of whole-body images indicated similar uptake in both kidneys, except for patient 3 (Table 2). Patient 4 was not examined because of logistical problems. Quantification of uptake in different regions at 2 and 24 hr is shown in Table 3. Greater uptake was observed in the liver and kidneys with similar biodistribution 2 and 24 hr after the cell infusion.

### 3.4. Laboratory Data

The patients underwent laboratory tests according to the initial planning (Table S1). To better assess kidney function, the results of the serum values for creatinine, urea, sodium, potassium, and urinary protein were compared with the average values for each patient during the follow-up (Table 4). Creatinine values differed between day −7 and day +2 and between day +2 and day +30. Further laboratory data were compared at −90 and +270 days, and no differences were found (Table S4).

## 4. Discussion

The present study evaluated the safety and biodistribution of infusion of autologous BMDMCs through renal arteries in patients with refractory FSGS over a 360-day period. All patients tolerated cell infusion without adverse clinical events and 1-year survival was 100%. A transient increase in creatinine level was observed after cell therapy, which was improved at 30 days and remained stable at the end of the study. ^99m^Tc-labeled BMDMCs were detected in both kidneys after 2 and 24 hr. The novelty of our work lies in the fact that we describe, for the first time, the biodistribution of radiolabeled autologous BMMCs after bilateral infusion into the kidneys. To our knowledge, there is only one study that used arterial infusion in both kidneys in chronic kidney disease [14], and none in patients with FSGS.

Preclinical studies have shown promising results in different models of kidney disease using autologous BMDMCs or mesenchymal stem cells (MSCs) [15]. Using the ischemia-reperfusion model, Semedo et al. [16] demonstrated a reduction in pro-fibrotic cytokines. such as IL-6 and TNF-*α*, and histologic markers of fibrosis 6 weeks after BMDMC infusion. Barreira et al. [17] showed reduced apoptosis and enhanced proliferation of tubular cells as well as reduced collagen and myofibroblasts in a model of unilateral ureteral obstruction followed by BMDMC therapy. Our group also showed benefits with BMDMCs in diabetic nephropathy [18] and ischemia-reperfusion injury [19]. Both types of cells present particular advantages. BMDMCs can be used in autologous transplantation on the day of harvesting, avoiding common complications such as graft-versus-host disease, whereas MSCs have multilineage differentiation potential and immune-privileged features that enable allogeneic use. So far, clinical studies have demonstrated the safety of systemic BMDMCs/MSCs infusion in kidney diseases, with no significant adverse effects reported [20]. Our decision to include patients with an eGFR of 20–40 ml/min/1.73 m^2^ was based on the premise of intervening before the onset of end-stage disease while excluding those at immediate risk for dialysis. Given the slow nature of CKD progression and the potential for variability in disease trajectory, a 12-month period provides an initial assessment of safety and potential signals of efficacy.

The intra-arterial route of administration has the potential to reach the organ directly with a high number of viable cells. In addition, it is effective in avoiding pulmonary entrapment mainly for MSCs [21]. Maximal therapeutic efficiency is more easily achieved but the high number of cells can cause hypoperfusion as a result of cell occlusion [22]. However, few studies have used intra-arterial cell infusion, probably because it is a more invasive procedure. In our study, four patients had a hematoma at the puncture site of the femoral artery and patient 3 presented an unknown anatomic variation in the left renal artery. Eco Color Doppler examinations of the renal arteries were performed in all patients to assess renal vascularization at −90 and +270 days and no anatomic changes were detected. The high prevalence of anatomic variations should be considered in future studies. It has been reported that accessory arteries are present in up to 30% of cases and are bilateral in 10% of patients. The use of EcoColor Doppler imaging uncovered previously undiagnosed renal artery abnormalities in 9%–23.5% of cases [23, 24]. Possible complications related to the endovascular procedure were analyzed in clinical trials of patients with renal artery stenosis undergoing angioplasty. The average occurrence rates and variation found in the different studies were excessive bleeding 2.9% (0.8%–16%), perforation or dissection of the renal artery 3.0% (1%–10%), thrombosis or obstruction of the renal artery 1.3% (0.4%–3.8%), and renal artery pseudoaneurysm 2.0% (0.3%–5.9%) [25]. Nevertheless, our procedure may be considered less complex due to the absence of angioplasty, but the rates of local complications may be similar.

Filgastrime (G-CSF) can mobilize hematopoietic stem cells and bone marrow progenitor cells to peripheral blood and is commonly used to facilitate collection of cells through apheresis before autologous bone marrow transplantation [26]. In our study, G-CSF was used to increase the supply of stem cells to injured tissues. Several animal experiments have shown beneficial effects of G-CSF therapy, including adriamycin nephropathy, by reductions in the number of atrophic sites, the severity of fibrosis, and the glomerulosclerosis rate [27]. However, clinical studies show inconclusive results on the benefit of this therapy. Side effects include bone pain, insomnia, and flu-like symptoms; these were reported in only one patient in our study [28].

Cells were labeled with ^99m^Tc to evaluate the biodistribution of the injected cells into the renal arteries based on previously published protocols [29, 30]. There are few clinical studies demonstrating the biodistribution of stem cells in vivo, and our study is the first to use infusion via the renal artery. Greater uptake was shown in the liver followed by the kidneys with decay after 24 hr. In a model of acute kidney injury, injection of MSCs into the suprarenal aorta showed distinct localization to the kidneys with a significant decline after 24 hr [31]. These results are similar to the data found in our study. A clinical trial to assess the biodistribution of ^99m^Tc-labeled BMDMCs delivered by different routes in patients with subacute middle cerebral artery ischemic stroke found no differences in renal uptake between intra-arterial and intravenous delivery but with higher signals after 24 hr compared with the signals at 2 hr [29]. Comparison between studies for the treatment of different pathologies has many limitations but a higher renal uptake after 2 hr in our study suggests that the renal arterial route may be more effective.

The analysis of the laboratory results showed a transient increase in creatinine levels, which was observed 48 hr after cell therapy and improvement in 30 days. A study with autologous MSCs infused in the renal artery for the treatment of renal artery stenosis showed an increase in the creatinine value after 24 hr of cell therapy, again with improvement in 30 days. We used a reduced medium dose of iodinated contrast (2.98 ml for each kidney), bicarbonate solution, and acetylcysteine to reduce contrast nephrotoxicity. Recent studies showed similar results using saline compared with bicarbonate solution. Low contrast doses appear to be beneficial in reducing contrast nephrotoxicity in coronary angiography in patients with GFR < 30 ml/min [32, 33]. There are no dose–response studies with the use of ^99m^Tc-labeled BMDMCs in renal arteries and the possibility of embolism cannot be ruled out. The analysis of laboratory tests between −90 days and +270 days was performed to identify possible changes related to the cell therapy or chronic kidney disease and showed no significant differences (Table 4).

Our study was an open-label, non-randomized pilot study, limited by a small sample size. Other limitations were the absence of genetic tests in patients with refractory FSGS and the potential beneficial effect of G-CSF therapy. This study was designed to examine safety, therefore the analysis was only observational, and further larger-scale trials are necessary to fully examine the efficacy of BMDMCs in patients with FSGS.

## 5. Conclusion

Based on this preliminary study, the administration of BMDMCs through renal arteries appears to be safe. Further clinical investigation of BMDMCs or even other cell types by different routes of infusion in patients with FSGS is warranted.

## Figures and Tables

**Table 1:** Demographic and clinical characteristics of the participants.

Clinical data	Patient 1	Patient 2	Patient 3	Patient 4	Patient 5
Age (years)	34	34	49	32	56
Sex	Female	Female	Female	Male	Male
Race	Black	White	White	White	White
Renal biopsy	FSGS	FSGS	FSGS	Collapsing FSGS	FSGS
Clinical presentation	Nephrotic syndrome	AKI	Nephrotic syndrome	Nephrotic syndrome	Nephrotic syndrome
Previous treatment	GCC	GCC,CP	GCC, CsA	GCC	GCC, CP
Other diseases	Migraine, HTN	HTN	HTN	HTN, nephrolithiasis	BPH, hypothyroidism, HTN
Date of infusion	29 Sep 2015	10 Nov 2015	8 Dec 2015	9 Mar 2016	16 May 2017
Creatinine (mg/dl)	1.76	2.40	2.00	2.20	2.38
UPCR (mg/g)	1701	1,252	214	1,450	3,340
eGFR (CKD-EPI) (ml/min/1.73 m^2^)	37.2	25.6	28.7	38.2	29.4
Use of ACEi/ARB	ACEi	ARB	ARB	ACEi	ACEi
Follow-up time (months)	12	12	12	12	12

Clinical data at baseline. FSGS, focal segmental glomerulosclerosis; AKI, acute kidney injury; GCC, glucocorticoid; CP, cyclophosphamide; CsA, cyclosporine; HTN, hypertension; BPH, benign prostatic hyperplasia; UPCR, urine protein-creatinine ratio; eGFR, estimated glomerular filtration rate; CKD-EPI, Chronic Kidney Disease Epidemiology Collaboration; ACEi, angiotensin-converting enzyme inhibitor; ARB, angiotensin receptor blocker.

**Table 2:** Quantification of uptake in whole-body planar images in kidneys at 2 and 24 hr after cell therapy (%).

Patients	RK 2 hr	LK 2 hr	RK 24 hr	LK 24 hr	TK 2 hr	TK 24 hr
1	3.5	2.0	4.5	1.9	5.5	6.4
2	12.0	8.0	7.7	3.3	20.0	11.0
3	10.0	3.0	6.0	1.0	13.0	7.0
4	3.5	4.6	—	—	8.1	—
5	10.0	9.0	5.0	2.9	19.0	7.9
Mean	7.80	5.32	5.80	2.28	13.12	8.08
SD	4.009	3.068	1.412	1.034	6.426	2.045

RK, right kidney; LK, left kidney; TK, total kidney; SD, standard deviation. Examination of patient 4 was not possible after 24 hr due to technical problems.

**Table 3:** Quantification of uptake in whole-body planar images in different regions at 2 and 24 hr after cell therapy (%).

Patient	Kidneys/whole body	Bladder/whole body	Liver/whole body	Lungs/whole body	Spleen/whole body	Brain/whole body
After 2 hr						
1	5.5	1.7	44.3	11.0	4.6	0.6
2	20.0	0.7	34.6	8.2	2.4	0.9
3	13.0	1.4	34.2	5.6	2.7	0.8
4	8.1	2.4	27.3	8.8	4.7	1.0
5	19.0	1.7	42.9	7.2	3.7	0.6
Mean	13.12	1.580	36.66	8.160	3.620	0.7800
±SD	6.426	0.6140	6.986	1.997	1.057	0.1789
After 24 hr						
1	6.4	0.4	41.1	3.4	4.5	0.8
2	11.0	1.3	32.0	7.3	3.6	0.9
3	7.0	0.6	22.4	2.6	1.8	1.3
4	—	—	—	—	—	—
5	7.9	1.2	35.0	3.4	4.2	1.2
Mean	8.075	0.8750	32.63	4.175	3.525	1.050
±SD	2.045	0.4425	7.798	2.117	1.209	0.2380

SD, standard deviation.

**Table 4:** Results of laboratory tests with the reference values.

Laboratory results	Reference value	−90 days	−7 days	+1 days	+2 days	+7 days	+15 days	+30 days	+90 days	+180 days	+270 days
Cr (mg/dl)	0.5–1.1	2.15 ± 0.27	2.09 ± 0.32^a^	2.58 ± 0.44	2.77 ± 0.4^ba^	2.22 ± 0.30	2.36 ± 0.32	2.09 ± 0.38^b^	2.23 ± 0.35	2.31 ± 0.53	2.35 ± 0.62
PCR (mg/g)	<200	1,450 (733–2,521)	565 (367–2,814)	153 (62–2,162)	158 (111–1,079)	215 (52–728)	564 (152–2,392)	860 (295–2,355)	777 (299–3,288)	1,359 (377–3,356)	861 (415–3,277)
Ur (mg/dl)	1550	69.8 ± 25.94	71.0 ± 14.44	79.6 ± 19.93	78.2 ± 16.12	71.0 ± 9.67	68.2 ± 22.98	63.8 ± 12.83	66.0 ± 16.03	70.8 ± 22.83	66.2 ± 17.17
Na^+^ (mEq/l)	138–145	140.0 ± 2.00	143.0 ± 1.87	140.8 ± 2.17	141.4 ± 1.52	142.6 ± 2.88	143.4 ± 2.79	142.4 ± 2.07	143.8 ± 2.59	143.0 ± 1.73	142.8 ± 3.42
K^+^ (mEq/l)	3.5–5.5	4.70 ± 0.23	4.82 ± 0.49	4.74 ± 0.70	4.74 ± 0.44	4.66 ± 0.43	4.78 ± 0.94	4.66 ± 0.62	4.52 ± 0.48	4.66 ± 0.52	4.46 ± 0.51

Laboratory data obtained from patients during the follow-up. Cr, creatinine; PCR, protein-creatinine ratio (urinary); Ur, urea; Na^+^, sodium; K^+^, potassium. Values are the mean ± standard deviation (SD) or median (interquartile range) of five patients per day. Repeated measures Friedman test followed by Dunn's multiple comparisons test were used (*p* < 0.05). Negative sign represents the number of days before therapy. ^a^*p* < 0.05 at −7 days versus +2 days. ^b^*p* < 0.05 at +2 days versus +30 days.

## Data Availability

The data that support the findings of this study are available from the corresponding author upon reasonable request.

## Supplementary Materials

Supplementary Materials The clinical events and treatments that occurred during follow-up, and further laboratory data can be found here.
