# Supplementary material for: Safety and Biodistribution of an Autologous Bone Marrow-Derived Mononuclear Cell Infusion into Renal Arteries in Patients with Focal Segmental Glomerulosclerosis: A Phase 1 Study
Source: Stem Cells Int. 2024 Jul 9;2024:2385568. doi: 10.1155/2024/2385568 (PMC11251782; doi:10.1155/2024/2385568)
Supplement: Supplementary Materials — The clinical events and treatments that occurred during follow-up, and further laboratory data can be found here. [file 2385568.f1.docx]

**Supplementary Material**

**Table S1.** Parameters evaluated during the study to analyze the cell therapy.

| **Evaluated parameters** | **Control time** | | | **CT** | **Follow-up time** | | | | | | | |
| --- | --- | --- | --- | --- | --- | --- | --- | --- | --- | --- | --- | --- |
| Days (±2) | −90 | −45 | −83 | 0 | +1 | +2 | +7 | +15 | +30 | +90 | +180 | +270 |
| Visit | 1 | 2 | 3 | 4 | 5 | 6 | 7 | 8 | 9 | 10 | 11 | 12 |
| Clinical evaluation | X | X | X | X | X | X | X | X | X | X | X | X |
| SF-36 questionnaire | X |  |  |  |  |  |  |  | X | X | X | X |
| Bone marrow culture |  |  |  | X |  |  |  |  |  |  |  |  |
| Urinalysis | X |  |  |  | X | X | X | X | X | X | X | X |
| Urine culture | X |  |  |  |  |  |  |  |  |  |  |  |
| PCR | X |  | X |  | X | X | X | X | X | X | X | X |
| Serum HCG | X^a^ |  |  |  |  |  |  |  |  |  |  |  |
| Urinary HCG |  |  |  | X^a^ |  |  |  |  |  |  |  |  |
| Lipidogram | X |  |  |  |  |  |  |  |  |  |  | X |
| PTH | X |  |  |  |  |  |  |  |  | X |  | X |
| 25-(OH)D | X |  |  |  |  |  |  |  |  | X |  | X |
| 1,25-(OH)_2_D | X |  |  |  |  |  |  |  |  | X |  | X |
| Calcium | X |  |  |  |  |  |  |  |  | X |  | X |
| Phosphorus | X |  |  |  |  |  |  |  |  | X |  | X |
| Glucose | X |  |  |  | X | X | X | X | X | X | X | X |
| Sodium | X |  | X |  | X | X | X | X | X | X | X | X |
| Potassium | X |  | X |  | X | X | X | X | X | X | X | X |
| Creatinine | X |  | X |  | X | X | X | X | X | X | X | X |
| BUN | X |  | X |  | X | X | X | X | X | X | X | X |
| Uric acid | X |  |  |  |  |  |  |  |  | X |  | X |
| ALT/AST | X |  |  |  |  |  |  |  |  |  |  | X |
| Coagulogram | X |  |  |  |  |  |  |  |  |  |  | X |
| Total protein and fractions | X |  |  |  |  |  |  |  |  | X |  | X |
| CRP | X |  |  | X | X | X | X | X | X | X |  | X |
| ESR | X |  |  | X | X | X | X | X | X | X |  | X |
| IL-6 | X |  |  |  |  | X | X |  | X | X | X | X |
| TNF-α | X |  |  |  |  | X | X |  | X | X | X | X |
| CBC | X |  | X | X | X |  | X | X | X | X | X | X |
| Iron | X |  |  |  |  |  |  |  |  | X |  | X |
| Ferritin | X |  |  |  |  |  |  |  |  | X |  | X |
| TSAT | X |  |  |  |  |  |  |  |  | X |  | X |
| Bicarbonate | X |  |  |  |  |  |  |  |  | X |  | X |
| ECG | X |  |  |  |  |  |  |  |  |  |  | X |
| TTE | X |  |  |  |  |  |  |  |  |  |  | X |
| CXR | X |  |  |  |  |  |  |  |  |  |  | X |
| US | X |  |  |  |  |  |  |  |  |  |  | X |
| DUS | X |  |  |  |  |  |  |  |  |  |  | X |

A negative sign represents the number of the days before cell infusion.

CT, cell therapy; PCR, protein-creatinine ratio (urinary); HCG, human chorionic gonadotropin; PTH, parathyroid hormone; BUN, blood urea nitrogen; ALT, alanine aminotransferase; AST, aspartate aminotransferase; CRP, C-reactive protein; ESR, erythrocyte sedimentation rate; IL-6, interleukin 6; TNF-α, tumor necrosis factor alpha; CBC, complete blood count; TSAT, transferrin saturation; ECG, eletrocardiogram; TTE, transthoracic echocardiogram; CXR, chest x-ray; US, ultrasound (abdominal and urinary tract); DUS, Doppler ultrasound (renal arteries).

^a^Female patients only.

**Table S2.** Description of the clinical events and treatments.

| **Patients** | **Time (days)** | **Clinical event** | **Treatment** |
| --- | --- | --- | --- |
| 1 | +180 | Cutaneous herpes | Topical medication |
| 2 | +180 | Pharyngitis | Azithromycin |
| 3 | +180 | Arbovirus infection | Spontaneous improvement |
| 4 | +270 | Acute renal colic | Symptomatic medication |
| 5 | +60 | Pyelonephritis | Ciprofloxacin |

A positive sign represents the number of days after cell infusion.

**
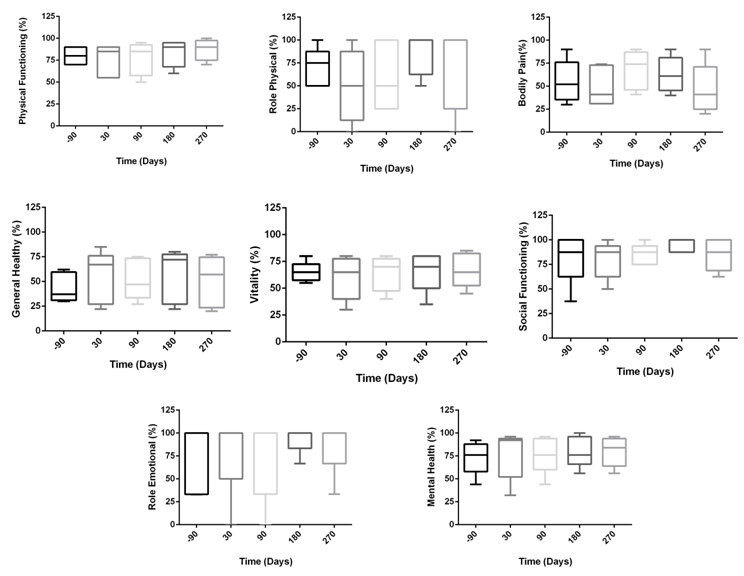
**

**Figure S1.** Evolution of the quality of life domains (physical functioning, role-physical, bodily pain, general health, vitality, social functioning, role emotional, mental health), as measured by the SF-36 questionnaire, during the 360-day follow-up period. All parameters remained stable throughout. Values are the median (25th–75th percentiles) of 5 patients at each time point. A negative sign represents the number of the days before cell infusion

**Table S3**. Composition of the cell suspensions and contrast volume.

| **Patient** | **No. of cells injected** | **BMDMC (%)** | **MSC (%)** | **HSC (%)** | **Viability (%)** | **Contrast volume (ml)** |
| --- | --- | --- | --- | --- | --- | --- |
| 1 | 5 × 10^7^ | 71.31 | 0.01 | 3.5 | 95.1 | 6.0 |
| 2 | 5 × 10^7^ | 71.9 | 0.02 | 2.7 | 94.8 | 6.0 |
| 3 | 5 × 10^7^ | 34.15 | 0.02 | 1.4 | 93.6 | 14.0 |
| 4 | 5 × 10^7^ | 53.6 | 0.02 | 1.5 | 94.1 | 2.4 |
| 5 | 5 × 10^7^ | 78.5 | 0.02 | 1.13 | 93.9 | 1.4 |
| Mean | 5 × 10^7^ | 61.89 | 0.0180 | 2.046 | 94.30 | 5.960 |
| ± SD | 0 | 18.05 | 0.00447 | 1.012 | 0.6285 | 4.953 |

The total number of cells infused by each kidney. BMDMC, bone marrow-derived mononuclear cells; MSC, mesenchymal stem cells; HSC, hematopoietic stem cells. Values are means ± standard deviation (SD).

**Table S4.** Results of the laboratory tests with the respective reference values.

| **Laboratory results (reference value)** | **−90 days** | **270 days after cell therapy** | ***p* value** |
| --- | --- | --- | --- |
| Creatinine (0.5–1.1 mg/dl) | 2.15 ± 0.27 | 2.35 ± 0.61 | 0.438 |
| PCR (<200 mg/g) | 1450 [733 to 2521] | 861 [415 to 3277] | 0.999 |
| BUN (15–50 mg/dl) | 69.8 ± 25.9 | 66.2 ± 17.2 | 0.999 |
| Sodium (138–145 mEq/l) | 140.0 ± 2.0 | 142.8 ± 3.4 | 0.125 |
| Potassium (3.5–5.5 mEq/l) | 4.70 ± 0.23 | 4.46 ± 0.51 | 0.438 |
| Uric acid (1.5–6.0 mg/dl) | 7.56 ± 1.56 | 7.42 ± 2.51 | 0.813 |
| Glucose (70–99 mg/dl) | 88.6 ± 10.85 | 83.4 ± 2.07 | 0.375 |
| Hematocrit (36%–46%) | 42 ± 3.39 | 43 ± 5.66 | 0.688 |
| Hemoglobin (12–16 g/dl) | 13.3 ± 1.43 | 13.7 ± 1.47 | 0.063 |
| Leukogram (4500–11,000/μl) | 10,240 [5385–12,470] | 7480 [5000–10,225] | 0.505 |
| Platelets (150,000–450,000/μl) | 228,500 [178,000–347,000] | 201,000 [108,250–449,000] | 0.625 |
| ESR (≤20 mm) | 2 [2–18.5] | 3 [2–49] | 0.999 |
| CRP (<5 mg /dl) | 1.8 [0.35–43.25] | 1.3 [0.35–2] | 0.438 |
| IL-6 (0.00–5.90 pg/ml) | 5.47 ± 6.32 | 2.69 ± 1.54 | 0.750 |
| TNF-α (<8.1 pg/ml) | 11.44 ± 3.23 | 9.8 ± 2.61 | 0.625 |
| Calcium (8.8–10.0 mg/dl) | 9.2 ± 0.55 | 9.2 ± 0.26 | 0.813 |
| Phosphorus (2.5–4.8 mg/dl) | 3.6 ± 0.36 | 3.2 ± 0.52 | 0.375 |
| PTH (12–65 pg/ml) | 38 [33 to 91.5] | 71 [51.5 to 135.5] | 0.063 |
| 25(OH)D (≥30 ng/ml) | 26.4 ± 9.1 | 28.0 ± 10.9 | 0.375 |
| 1.25(OH)_2_D (18–78pg/ml) | 46.6 ± 18.7 | 31.8 ± 10.1 | 0.063 |
| Albumin (3.2–4.5 g/dl) | 4.1 ± 0.21 | 4.3 ± 0.27 | 0.250 |
| Bicarbonate (22–28 mEq/l) | 26.6 ± 2.70 | 26.2 ± 2.78 | 0.688 |
| ALT (7–35 U/l) | 14.4 ± 5.94 | 17.6 ± 11.06 | 0.438 |
| AST (13–35 U/l) | 18.2 ± 6.14 | 21.2 ± 6.83 | 0.250 |
| Iron ((37–145 μg/ml) | 60.0 ± 32.73 | 61.4 ± 28.85 | 0.875 |
| Ferritin (10–291 ng/ml) | 84.8 ± 11.67 | 58.8 ± 30.85 | 0.250 |
| TSAT (15%–50%) | 26 [7.4 to 28] | 22 [10.75 to 43] | 0.625 |
| Cholesterol (<200 mg/dl) | 176 [162.5 to 188] | 203 [132.5 to 263.5] | 0.438 |
| Triglycerides (<150 mg/dl) | 195 [87 to 224.5] | 258 [193 to 296.5] | 0.063 |
| INR (0.8–1.2) | 1.08 ± 0.06 | 1.04 ± 0.05 | 0.313 |
| aPTT (<1.25 s) | 1.15 ± 0.12 | 1.07 ± 0.13 | 0.625 |

The values ​​are means ± standard deviation (SD) or medians [interquartile range] of five patients per day. The paired Wilcoxon test was used for statistical analysis (*p* < 0.05). A negative sign represents the number of days before cell therapy (CT). PCR, protein-creatinine ratio (urinary); PTH, parathyroid hormone; BUN, blood urea nitrogen; ALT, alanine aminotransferase; AST, aspartate aminotransferase; CRP, C-reactive protein; ESR, erythrocyte sedimentation rate; IL-6, interleukin 6; TNF α, tumor necrosis dactor alpha; CBC, complete blood count; TSAT, transferrin saturation; INR, international normalized ratio; aPTT, activated partial thromboplastin time. The platelet score for patient 4 was lost due to a clot in the sample (−90 days).


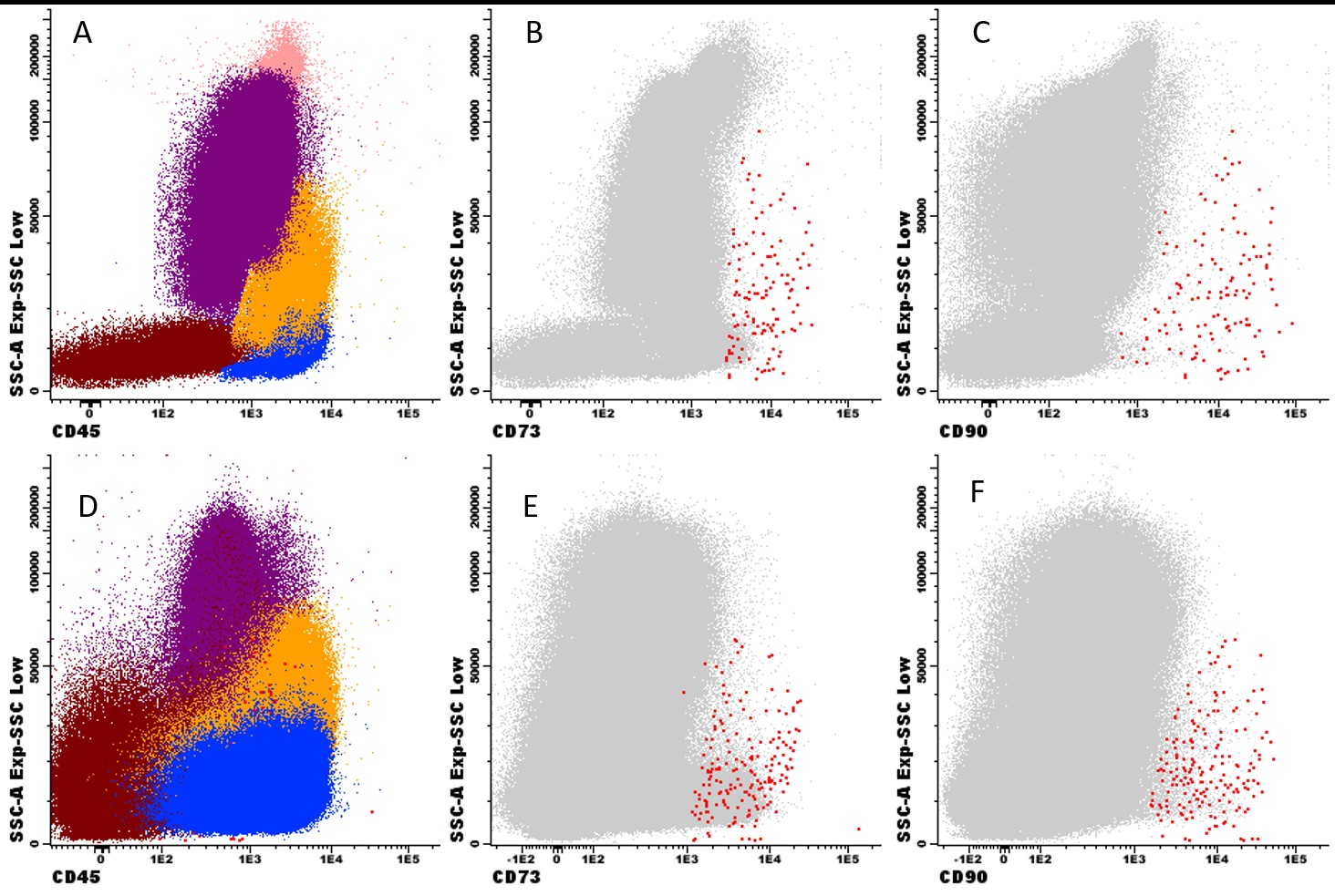


**Figure S2: Illustration of the gating strategy used for identification of mesenchymal stromal cells and Bone marrow populations:** A, B and C panel shows an illustrative example of gating strategy to identify bone marrow cells and D, E and F bone marrow-derived mononuclear cells (BMDMCs). Being in Red dots: Mesenchymal stromal cells; Blue dots: Total lymphocytes; Yellow dots: Total monocytes; Purple dots: Neutrophils and Dark red dots: Erythroblasts; Pink dots: Eosinophils; Grey dots: Bone marrow and BMDMCs cells without identification.

| Patients | HSC | Promonocytes | MSC | Erythroblasts | Monocytes | T Lymphocytes | Helper T cells | Cytotoxic T cells | B cells | | NK cells |
| --- | --- | --- | --- | --- | --- | --- | --- | --- | --- | --- | --- |
| 1 | 0.8 | 1.4 | 0.01 | 9.9 | 4 | 4.8 | 2.2 | 2.2 | | 1.7 | 0.4 |
| 2 | 0.9 | 1.5 | 0.01 | 7.3 | 4.6 | 10.1 | 5.2 | 4.2 | | 5.7 | 2.9 |
| 3 | 0.8 | 1.2 | 0.016 | 12.3 | 2.8 | 7.1 | 3.6 | 3.2 | | 2.1 | 0.8 |
| 4 | 1.3 | 1 | 0.013 | 7.2 | 4.9 | 7.3 | 3 | 4.1 | | 2.7 | 1.8 |
| 5 | 0.6 | 1 | 0.018 | 9.9 | 4.4 | 10.7 | 5.9 | 3.9 | | 2.2 | 1.6 |
| Mean | 0.9 | 1.2 | 0.013 | 9.3 | 2.9 | 7.9 | 3.9 | 3.5 | | 2.9 | 1.5 |
| SD | 0.2 | 0.2 | 0.003 | 1.9 | 0.8 | 2.2 | 1.4 | 0.7 | | 1.4 | 0.7 |

**Table S5. Flow cytometry individual analysis of total bone marrow cells**

HCS, Hematopoietic stem cells; MSC, Mesenchymal stromal cells; SD, standard deviation.

**Table S6. Flow cytometry individual analysis of BMDMCs**

| Patients | HSC | Promonocytes | MSC | Erythroblasts | Monocytes | T Lymphocytes | Helper T cells | Cytotoxic T cells | B cells | | NK cells |
| --- | --- | --- | --- | --- | --- | --- | --- | --- | --- | --- | --- |
| 1 | 3.5 | 4.8 | 0.01 | 2.7 | 21.5 | 42.8 | 20.8 | 20.2 | | 7.6 | 1.1 |
| 2 | 1.3 | 1 | 0.013 | 1.7 | 4.9 | 7.3 | 3 | 4.1 | | 2.7 | 1.8 |
| 3 | 1.4 | 1.9 | 0.015 | 3.0 | 7.7 | 15.7 | 8.1 | 7 | | 3.2 | 3.1 |
| 4 | 1.5 | 1.4 | 0.02 | 2.0 | 9.2 | 26.9 | 11 | 14.4 | | 8 | 9.5 |
| 5 | 1.1 | 1.6 | 0.02 | 5.1 | 9.8 | 61.2 | 29.4 | 24.3 | | 4.3 | 2.1 |
| Mean | 1.8 | 2.1 | 0.016 | 2.9 | 10.6 | 30.8 | 14.5 | 14 | | 5.2 | 3.5 |
| SD | 0.9 | 1.3 | 0.003 | 1.2 | 5.7 | 19.3 | 9.45 | 7.6 | | 2.2 | 3.0 |

HCS, Hematopoietic stem cells; MSC, Mesenchymal stromal cells; SD, standard deviation.
